# Analysis of the associations among *Helicobacter pylori* infection, adiponectin, leptin, and 10-year fracture risk using the fracture risk assessment tool: A cross-sectional community-based study

**DOI:** 10.1371/journal.pone.0175365

**Published:** 2017-04-07

**Authors:** Li-Wei Chen, Fang-Ping Chen, Chia-Wen Hsieh, Sheng-Fong Kuo, Rong-Nan Chien

**Affiliations:** 1 Department of Gastroenterology and Hepatology, Chang-Gung Memorial Hospital and University at Keelung, Keelung, Taiwan; 2 Community Medicine Research Center, Chang-Gung Memorial Hospital and University at Keelung, Keelung, Taiwan; 3 Department of Obstetrics and Gynecology, Chang-Gung Memorial Hospital at Keelung, Keelung, Taiwan; 4 Metabolism and Endocrinology, Chang-Gung Memorial Hospital and University at Keelung, Keelung, Taiwan; Van Andel Institute, UNITED STATES

## Abstract

*Helicobacter pylori* (*H*. *pylori*) infection may induce inflammatory cytokines or adipokines that influence bone turnover and bone fracture risk. This study aimed to evaluate the association among *H*. *pylori* infection, adipokines, and 10-year fracture risk using the Fracture Risk Assessment Tool scale. From August 2013 to February 2016, a community-based cohort was surveyed by Keelung Chang-Gung Memorial Hospital. Subjects were included if they were older than 40 years and not pregnant. All participants underwent a standardized questionnaire survey, physical examination, urea breath test, and blood tests. A total of 2,689 participants (1,792 women) were included in this cross-sectional study. In both sexes, participants with a high fracture risk were older and had higher adiponectin values than participants without a high fracture risk (mean age, female: 72.9 ± 5.6 vs. 55.8 ± 7.3 years, *P <* 0.0001; male: 78.9 ± 4.7 vs. 58.1 ± 8.9 years, *P <* 0.001) (adiponectin, female: 10.8 ± 6.3 vs. 8.7 ± 5.2 ng/ml, *P <* 0.001; male: 9.7 ± 6.1 vs. 5.5 ± 3.8 ng/ml, *P <* 0.001). Adiponectin was correlated with high fracture risk in both sexes, but *H*. *pylori* infection and leptin was not. In logistic regression analysis, adiponectin could not predict high fracture risk when adjusting the factor of body mass index (BMI) in men group. In conclusion, *H*. *pylori* infection and leptin could not predict 10-year fracture risk in either sex. Adiponectin was correlated with bone fracture risk in both sexes and the correlation might be from the influence of BMI.

## Introduction

Osteoporosis is a condition with progressively decreasing bone mineral density (BMD) and increased bone fragility, with increased risk of bone fractures [1,2]. The common sites of fractures are the spine and hip. Spine and hip fractures caused by osteoporosis are the primary factors responsible for bedbound status and the need for nursing care in the elderly [1,2]. To prevent bone fracture–related comorbidity, it is important to identify patients with underlying diseases or risk factors for osteoporosis and bone fractures. Some well-known risk factors for bone fracture include advancing age, postmenopausal status, low body weight, smoking, excess alcohol consumption, and previous fracture history [3–5]. Some digestive diseases, including inflammatory bowel disease and history of gastrectomy, are also reported to be risk factors for osteoporosis [6–8]. *Helicobacter pylori* (*H*. *pylori*) is a spiral-shaped, Gram-negative bacterium that colonizes the gastric mucosal epithelium. Most patients with *H*. *pylori* infection will develop chronic gastritis, which induces inflammatory cytokines, such as interleukin 1 (IL-1) and tumor necrosis factor alpha (TNF-α). These inflammatory cytokines induce both local and systemic immune responses [9–11]. Adipokines, such as serum adiponectin or leptin, are reportedly associated with the status of *H*. *pylori* infection [12–14]. Previous studies have reported that IL-1 and TNF-α trigger bone resorption [15–17]. Adipocytes and osteoblasts have the same mesenchymal stem cell precursors. Hence, adipocytes and osteoblasts share some similar hormones and aspects of cytokine regulation [18, 19]. Past studies have revealed a positive correlation between body fat mass and BMD [18–20]. Osteoporosis develops more often in people with low body weight but less often in obese people [3, 20]. Both adiponectin and leptin have been found to increase osteoblast proliferation and inhibit osteoclast activity [18–21]. Our hypothesis was that *H*. *pylori* infection might induce serum inflammatory changes in cytokines (IL-1 and TNF-α) or adipokines (adiponectin and leptin) that influence bone turnover and bone fracture risk. Prior epidemiological studies analyzing the association between *H*. *pylori* and osteoporosis showed conflicting results, and most studies had no inflammatory cytokine data [22–29]. If *H*. *pylori* infection could be a predictor of bone fracture, giving *H*. *pylori*–eradication therapy to prevent bone fracture would be warranted. Although the assay of BMD by dual-energy X-ray absorptiometry is the most common tool used to evaluate bone strength, the use of dual-energy X-ray absorptiometry to predict high risk of bone fracture is limited in mass community screening [30]. The World Health Organization (WHO) has developed a web-based clinical scale, the Fracture Risk Assessment Tool (FRAX), which predicts the 10-year probability of hip or other major osteoporotic fracture [31–33]. The FRAX estimate of risk of fracture is based on the assessment of readily accessible data regarding 10 clinical risk factors for fracture and can be obtained without BMD data [30–36]. Hence, this study aims to evaluate the association between *H*. *pylori* infection and 10-year fracture risk using the FRAX scale for 3 communities in northeastern Taiwan. Serum inflammatory cytokines and adipokines were also included in the analyses.

## Materials and methods

From August 2013 to February 2016, a community-based survey for metabolic syndrome was performed in 3 districts (Wanli, Ruifang, and Anle) in the northeastern region of Taiwan. A cross-sectional analysis from this cohort was performed. Subjects were included if they were older than 40 years and were not pregnant. All participants completed a demographic survey, physical examination, urea breath test (UBT), and blood tests. A standardized questionnaire was administered to all participants by a trained team of interviewers. The content of the questionnaire included a survey for systemic diseases, such as diabetes mellitus (DM), hypertension, hyperlipidemia, or chronic kidney disease; alcohol consumption and smoking; medication history (oral hypoglycemic agents, vitamin D or calcium supplementation, statins, herbal medicine, hormones or steroid usage, and antibiotics); and family history (malignancy or bone fracture history). The definition of tobacco use was “participants who currently smoke regardless of the number of cigarette packs.” Excessive alcohol was defined as “men drinking more than 30 g of alcohol and women drinking more than 20 g of alcohol daily.” Secondary osteoporosis was defined as osteoporosis related (or secondary) to diseases such as type I (insulin-dependent) diabetes, untreated long-standing hyperthyroidism, hypogonadism, premature menopause (<45 years), chronic malnutrition or malabsorption, and chronic liver disease. These diseases were surveyed by our standard questionnaire. Because this study focused on the association between *H*. *pylori* infection and bone fracture risk, those who took glucocorticoids, hormone replacement therapy, thyroid/parathyroid drugs, selective estrogen receptor modulators, vitamin D, calcium, bisphosphonates, and proton pump inhibitors were excluded. Those with comorbidity such as history of gastrectomy, inflammatory bowel disease, chronic obstructive pulmonary disease, malignant disease, thyroid or parathyroid disorder, and rheumatoid arthritis were also excluded. The Institutional Review Board of the Chang-Gung Memorial Hospital approved this research (IRB No. 102-2827C, 103-2392C1). Written informed consent was obtained from all subjects before enrollment in this study.

### Body Mass Index (BMI)

BMI was calculated as weight (kg) divided by height (meters) squared (kg/m^2^).

### Blood tests

Blood samples were collected from all participants after overnight fasting and were analyzed within 4 hours after collection for complete blood cell count and biochemistry tests. Adiponectin, leptin and TNF-α levels were determined from additional samples that were stored at -80°C after centrifugation (4°C at 3,000 rpm for 30 min).

### Bone fracture risk assessment

The FRAX tool without BMD was applied for bone fracture risk assessment (10-year probability of hip or other major osteoporotic fracture). The FRAX is based on the assessment of clinical risk factors, including age, individual and family history of fracture, tobacco use, excessive alcohol consumption, glucocorticoid use, body weight, secondary osteoporosis, and rheumatoid arthritis. There is consensus that people with a 10-year probability of hip fracture ≥3% or a 10-year probability of major osteoporotic fracture ≥20% by FRAX evaluation may be considered to have a high risk of bone fracture and a possible diagnosis of osteoporosis [34]. Local validation revealed good efficiency for bone fracture risk evaluation in postmenopausal women [35].

### Urea breath test

^13^C-UBT was performed using the Proto Pylori kit (Isodiagnostika, Montreal, Quebec, Canada), with local validation. Results were expressed as delta over baseline (DOB). A local validation test with a DOB cutoff value of 3.5 yielded a sensitivity of 95% and a specificity of 93% [37]. The result of the UBT was recognized as category data (positive or negative) in the regression analysis.

### Adiponectin and leptin

The assays for adiponectin and leptin employed quantitative sandwich enzyme immunoassay technique and were performed according to the manufacturer’s instructions (Human Total Adiponectin/Acrp30, BioVendor Research and Diagnostic system, Minneapolis, MN, USA; Human Leptin enzyme-linked immunosorbent assay, Clinical Range, BioVendor Laboratory Medicine, Karasek, Czech Republic). According to the manufacturer’s reference, the normal range of adiponectin value was ≧4.6 μg/ml in women and ≧2.04 μg/ml in men. The normal range of leptin value was ≦16.5 ng/ml in women and ≦7 ng/ml in men [38,39].

### Tumor necrosis factor α

The TNF-α assay used a quantitative sandwich enzyme immunoassay technique and was performed according to the manufacturer’s instructions (Immunite 1000 LKNF1; Siemens Medical Solutions Diagnostics, Lanberis, UK). The normal range of TNF-α value was less than 8.1 pg/ml, according to manufacturer’s reference [40].

### Statistical analysis

For continuous variables, values were expressed as means and standard deviations (SD). The chi-square test or Fisher’s exact test were applied for categorical data analysis. All statistical tests were 2-tailed, and *P* < 0.05 indicates a statistically significant difference. By maximizing Youden’s index and receiver operating characteristic (ROC) curve analysis, an optimal cutoff point of age to predict high 10-year fracture risk could be evaluated. Using the continuous variable of 10-year hip fracture probability (%) by FRAX assessment and age period, a lifetime bone fracture probability curve was created by dividing the participants into those with and without *H*. *pylor*i infection. The associations among *H*. *pylori* infection (evaluated by UBT), TNF-α, adiponectin, leptin, and bone fracture risk (evaluated by FRAX scale) were evaluated using Pearson’s or Spearman’s correlation coefficients. Phi coefficient was applied for binary data, such as *H*. *pylori* infection; Spearman’s coefficient rho was applied for a nonparametric measure of rank correlation, such as bone fracture risk; Pearson’s correlation was applied for continuous data, such as high-sensitivity C-reactive protein, TNF-α, adiponectin, and leptin. Because people with high bone fracture risk by FRAX evaluation were older and had lower BMI, multivariate logistic regression analysis was performed after adjusting for potential confounders of age and BMI. Other risk factors of smoking and excess alcohol consumption were also adjusted for bone fracture assessment by logistic regression analysis. SPSS for Windows software (Version 16.0, SPSS Inc., Chicago, IL, USA) was used for analysis.

## Results

**Fig 1:**
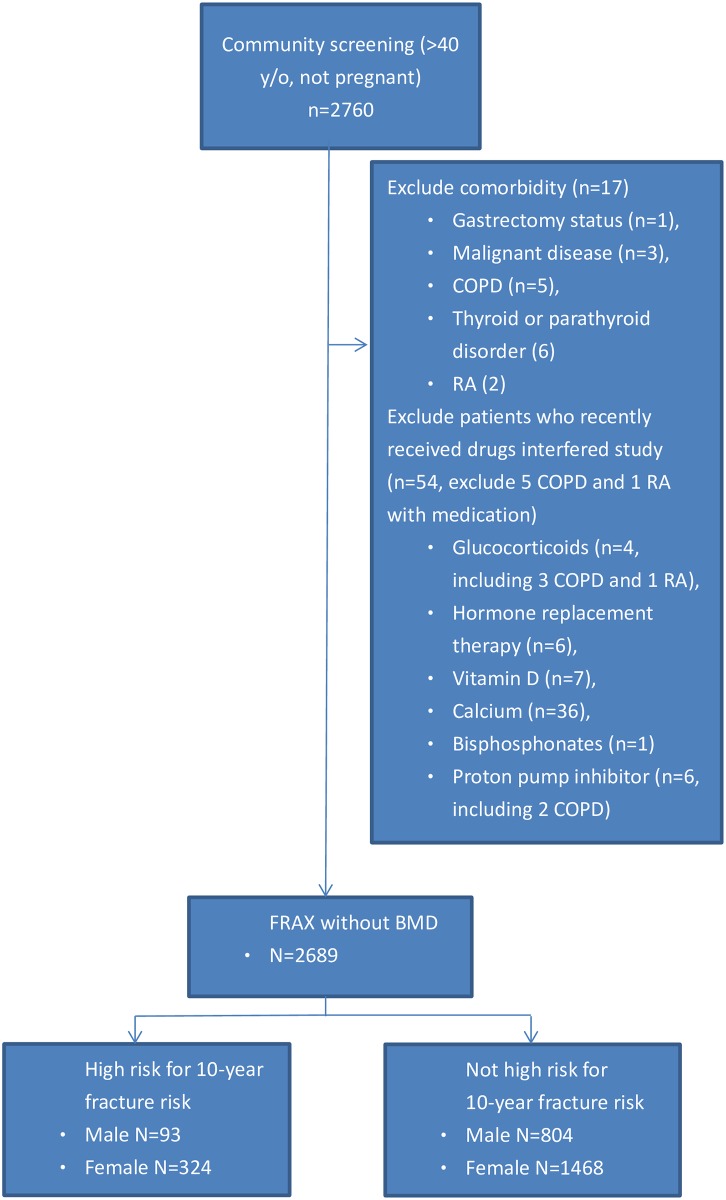
A flow diagram of participants. There were five participants with chronic obstructive pulmonary disease (COPD) and two participants with rheumatoid arthritis (RA). Three participants with COPD and 1 with rheumatoid arthritis who took steroid for disease control. Two participants with COPD took PPI for chronic cough possibly related to gastroesophageal reflux disease (GERD). Another one patient with RA did not take steroid.

Initially, 2,760 participants were enrolled. Finally, a total of 2,689 participants (897 men and 1,792 women) were included in this study (Fig 1, flowchart). The demographic data list was divided into 2 groups according to sex (Table 1). The percentage of individuals with high fracture risk was greater in women compared to men (324/1,792, 18.1%, vs. 93/897, 10.4%, *P <* 0.001). According to the questionnaire, 188 women (10.5%, 188/1,792) and 92 men (10.3%, 92/897) reported gastrointestinal symptoms. The usual symptoms were epigastric pain (82%), abdominal fullness (34%), and gastroesophageal reflux disease (18%). There was no statistical difference in the distribution of gastrointestinal symptoms between participants with and without high fracture risk in both sexes.

**Table 1:** Demography and characteristics of participants with and without high risk of 10-year bone fracture probability by sexes.

	Male			Female		
	High risk for fracture (-)	High risk for fracture (+)	*P* value^a^	High risk for fracture (-)	High risk for fracture (+)	*P* value^a^
Number (%)^b^	804 (89.6)	93 (10.4)		1,468 (81.9)	324 (18.1)	<0.001
Major osteoporotic fracture (%)^c^	4.1 ± 1.9	10.1 ± 4.0	<0.001	4.9 ± 2.3	13.7 ± 3.1	<0.001
Hip fracture (%)^c^	0.8 ± 0.8	4.9 ± 2.2	<0.001	1.0 ± 0.8	5.9 ± 2.3	<0.001
Mean age (years)	58.1 ± 8.9	78.9 ± 4.7	<0.001	55.8 ± 7.3	72.9 ± 5.6	<0.001
BMI	25.6 ± 3.5	23.7 ± 2.8	<0.001	24.4 ± 3.7	24.1 ± 3.6	0.092
Alcohol (%)	102 (12.7)	3 (3.2)	0.007	35 (2.4)	0 (0)	0.005
Smoking (%)	405 (50.4)	46 (49.5)	0.868	103 (7.0)	11 (3.4)	0.016
UBT (*H*. *pylori* %)	440 (54.7)	54 (58.1)	0.540	784 (53.4)	177 (54.6)	0.689
HS-CRP	2.3 ± 6.2	3.7 ± 11.9	0.042	2.0 ± 4.5	2.4 ± 8.0	0.299
TNF-α	8.2 ± 5.8	8.7 ± 4.3	0.231	7.4 ± 4.6	8.8 ± 9.1	<0.001
Adiponectin	5.5 ± 3.8	9.7 ± 6.1	<0.001	8.7 ± 5.2	10.8 ± 6.3	<0.001
Leptin	8.1 ± 6.5	7.7 ± 5.8	0.699	15.3 ± 8.7	14.7 ± 9.2	0.773
Calcium	9.4 ± 0.3	9.2 ± 0.3	<0.001	9.4 ± 0.3	9.4 ± 0.3	0.925

^a^ *P* value: categorical data (*H*. *pylori* %), chi-square test; continuous data, t test.

^b^ Comparison of the distribution percentage of high fracture risk between men and women.

^c^ The 10-year bone fracture probability using the Fracture Risk Assessment Tool. BMI = body mass index (kg/m^2^); HS-CRP = high-sensitivity C-reactive protein; TNF-α = tumor necrosis factor alpha; UBT (*H*. *pylori*) = urea breath test.

In women, participants with a high 10-year fracture risk were older and had higher adiponectin values than participants with a low 10-year fracture risk (mean age, 72.9 ± 5.6 vs. 55.8 ± 7.3 years, *P <* 0.001; adiponectin, 10.8 ± 6.3 vs. 8.7 ± 5.2 ng/ml, *P <* 0.001). However, the distribution of *H*. *pylori* infection (UBT-positivity) was not significantly different between the high and low 10-year fracture risk participants (54.6% vs. 53.4%, *P* = 0.689).

In men, the participants with a high 10-year fracture risk were also older and higher adiponectin values, than participants without a high 10-year fracture risk (mean age, 78.9 ± 4.7 vs. 58.1 ± 8.9 years, *P <* 0.001; adiponectin, 9.7 ± 6.1 vs. 5.5 ± 3.8 ng/ml, *P <* 0.001). The distribution of *H*. *pylori* infection (UBT-positivity) was also not significantly different between the high and low 10-year fracture risk participants (58.1% vs. 54.7%, *P* = 0.540).

The mean serum calcium value was lower in male participants with high fracture risk than in those without high risk (9.2 ± 0.3 vs. 9.4 ± 0.3, *P <* 0.001). However, there was no significant difference in mean serum calcium value between female participants with high fracture risk and those without high fracture risk. Among participants with and without *H*. *pylori* infection, the mean serum calcium values showed no significant difference.

**Fig 2:**
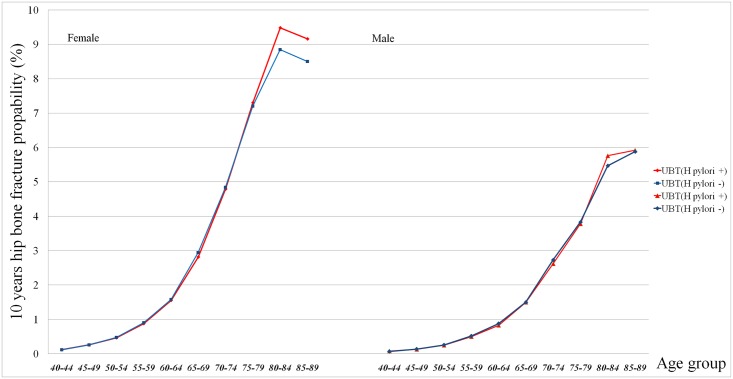
Lifetime bone fracture risk by the FRAX hip fracture probability (%) in participants with or without *H*. *pylori* infection. The numbers on the X-axis indicate the age interval (every 5 years) from 40 to 90 years old. The numbers on the Y-axis indicate the 10-year hip bone fracture probability (%) by FRAX. There was no significant difference in mean hip fracture probability (%) between participants with and without *H*. *pylori* infection in both sexes.

By maximizing Youden’s index and ROC curve analysis, the optimal cutoff point of age to predict high 10-year fracture risk was 65.5 years in all participants (sensitivity, 95.0%; specificity, 87.3%). Using the 10-year hip fracture probability (%) by FRAX assessment and age period, Fig 2 revealed a lifetime bone fracture probability curve by dividing the participants into those with and without *H*. *pylori* infection. There was no significant difference of hip fracture probability in every age period between participants with and without *H*. *pylori* infection. In both sexes, a rapidly increasing fracture risk by hip fracture probability >3% was found in participants older than 65 years.

Bivariate correlations and multivariate logistic regression analysis between the 10-year fracture risk and risk factors, such as age, BMI, smoking, excess alcohol consumption, *H*. *pylori* infection, and adiponectin, leptin, TNF-α, and high-sensitivity C-reactive protein levels, are shown in Table 2 (correlation analysis) and Table 3 (logistic regression analysis). *H*. *pylori* infection and leptin were not correlated with bone fracture risk in both sexes. Adiponectin values were positively correlated with fracture risk in both sexes (*P* < 0.05). However, when adjusting the confounding factor of BMI, there was no statistical difference of adiponectin value for the odds ratio of high fracture risk in men group by multivariate logistic regression. There may be an existence of co-linearity between adiponectin and BMI in this regression analysis.

**Table 2:** Bivariate correlation between factors and high fracture risk.

Factor^a^	Men	Women
Age	0.598*	0.684*
BMI	-0.163*	-0.040
Alcohol	-0.090*	-0.066*
Smoking	-0.006	-0.057*
*H*. *pylori*	0.020	0.009
HS-CRP	0.012	0.034
TNF-α	0.028	0.096*
Adiponectin	0.298*	0.148*
Leptin	-0.016	-0.025

^a^ Phi coefficient analysis for binary category data (*H*. *pylori*) and Spearman’s coefficient rho for rank correlation (high bone fracture risk) and Pearson’s correlation coefficient for continuous data (HS-CRP, TNF-α, adiponectin, leptin). BMI = body mass index; HS-CRP = high-sensitivity C-reactive protein; TNF-α = tumor necrosis factor alpha;

*P < 0.05.

**Table 3:** Multivariate logistic regression between factors and high fracture risk.

Factor	Multivariate regression					
Men	Model 1		Model 2		Model 3	
	OR (95% CI)	*P*	OR (95% CI)	*P*	OR (95% CI)	*P*
Age					2.131(1.701–2.670)	<0.001
BMI					0.735(0.598–0.904)	0.003
Alcohol			0.247(0.075–0.809)	0.021	1.749(0.158–19.306)	0.648
Adiponectin	1.185(1.133–1.240)	<0.001	1.184(1.131–1.239)	<0.001	1.063(0.968–1.168)	0.201
Women						
Age					1.760(1.622–1.910)	<0.001
Smoking			0.549(0.279–1.079)	0.549	2.301(0.755–7.011)	0.143
TNF-α	1.040(1.008–1.074)	0.014	1.040(1.007–1.073)	1.040	1.005(0.937–1.078)	0.883
Adiponectin	1.064(1.040–1.089)	<0.001	1.063(1.039–1.088)	<0.001	1.052(1.009–1.098)	0.019

OR = odd ratio; 95% CI = 95% confidence interval.

Factors correlated with bone fracture risk were entering the logistic regression analysis. These factors were age, BMI, adiponectin and alcohol for men group and age, smoking, adiponectin and TNF-α for women group. Although the factor of alcohol was correlated with bone fracture risk in women group, alcohol was not entering the regression analysis in women group. The reason was no women in high fracture risk reported to drink excess alcohol (N = 0). It may be not necessary to enter alcohol into adjustment for the regression analysis in women group. Hence, according to logistic regression analysis to estimate odds for high 10-year fracture risk with adjustments for age, smoking and excess alcohol consumption, adiponectin could predict bone fracture risk in both sexes. However, adiponectin losses the predictor role for bone fracture risk when entering the factor of BMI into adjustment in men group. An existence of co-linearity between adiponectin and BMI was found in this regression analysis for bone fracture risk.

**Fig 3:**
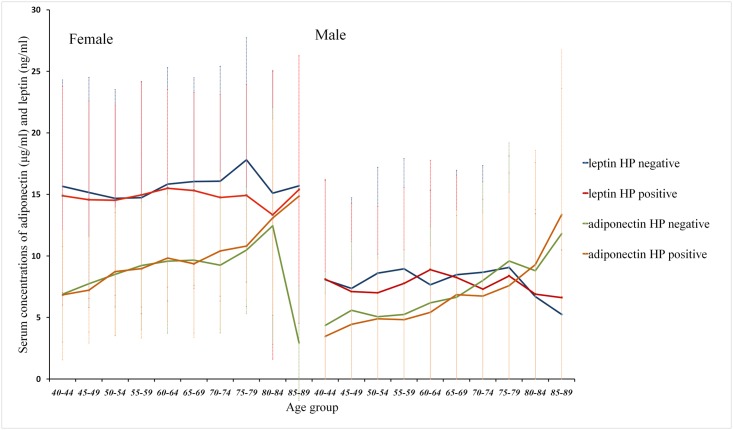
Mean values of adiponectin and leptin in participants with or without *H*. *pylori* infection. The numbers on the X-axis indicate the age interval (every 5 years) from 40 to 90 years old. The numbers on Y-axis axis indicate the serum concentrations of adiponectin (μg/ml) and leptin (ng/ml). The mean serum adiponectin values increased with increased ages of participants. The mean adiponectin and leptin values were higher in women than in men. There was no significant difference in mean adiponectin and leptin values between participants with and without *H*. *pylori* infection in any age period.

Fig 3 shows the mean adiponectin and leptin values in individuals with or without *H*. *pylori* infection by age stratification. The mean adiponectin values were increased in older participants. The mean adiponectin and leptin values were higher in women than in men. In every age group, there was no significant difference in mean adiponectin and leptin values between participants with and without *H*. *pylori* infection. The correlation and regression analysis revealed no association between adiponectin/leptin values and *H*. *pylori* infection in this study.

## Discussions

Past studies about the association between *H*. *pylori* infection and osteoporosis or fracture risk are shown in Table 4. Four studies reported a positive association between *H*. *pylori* infection and osteoporosis [22,27–29]. Figura et al. [22] reported that male patients infected by CagA protein–producing *H*. *pylori* strains had higher values of a urinary bone fracture biomarker. Two hospital-based studies from Japan by Asaoka et al. [27,29] and 1 from Taiwan by Lin et al. [28] reported an association between *H*. *pylori* infection and osteoporosis. Subjects in a hospital-based group may include symptomatic patients with more severe inflammatory conditions, such as erosive gastritis, peptic ulcers, and gastric mucosal atrophy [27–29]. Although there was no detailed information about the serum calcium, inflammatory cytokine, or estrogen level in these 3 studies, the authors tried to explain the association with *H*. *pylori* infection-related diseases and osteoporosis based on 3 reasons. The first reason was that gastric mucosal atrophy related to *H*. *pylori* infection might induce malabsorption of calcium, which plays a crucial role in mineral homeostasis and bone metabolism [25]. The second reason was that systemic inflammatory cytokines related to *H*. *pylori* infection interfered with the differentiation of osteoclasts, which are related to bone destruction [11,15]. The third reason was that patients with CagA-expressing *H*. *pylori* strains had a lower serum estrogen level than those infected by non-CagA-expressing strains and normal controls of both sexes [22]. Estrogen deficiency related to CagA-positive *H*. *pylori* infection was the reason for increasing bone resorption and fracture risk [22,41,42]. In our study, the majority of subjects had no symptom of gastrointestinal disease. Our study revealed no significant differences in *H*. *pylori* infection distribution between people with and without a high risk of fracture in either sex. Moreover, the mean serum calcium value was not significantly different between those with and without *H*. *pylori* infection. We could not confirm if the positive association between *H*. *pylori* infection and osteoporosis would only occur in symptomatic patients because most subjects in our study were asymptomatic. Further studies to elucidate the association between *H*. *pylori* infection and bone fracture risk are warrant by including more patients with *H*. *pylori* related diseases, such as atrophic gastritis or increasing serum inflammatory cytokines.

**Table 4:** Recent studies about the association between *H*. *pylori* and Osteoporosis/Bone fracture risk.

Year/nation/first author	Sex/age (years) (number)^a^	*H*. *pylori* detection	Bone fracture risk survey	Association	Ref
2005/Italy/Figura	Male/65 (80/160)	Serum antibody	Urinary biomarker	Positive in CagA (+) strain	[22]
2007/Brazil/Kakebasi	Female/61.7 (50/0)	C13 UBT/RUT/histology	DEXA for osteoporosis	No association	[23]
2007/Turkey/Ozdem	Both/11.8 (41/20)	RUT/histology	Biochemical markers	No association	[24]
2009/Brazil/Kakebasi	Female/63.7 (34/0)	Histology/UBT	DEXA for osteoporosis	No association	[25]
2011/Turkey/Akkaya	Female/65.3 (58/47)	Serum antibody	DEXA for osteoporosis	No association	[26]
2014/Japan/Asaoka	Both/63.1 (41/159)	C13 UBT/serum antibody	DEXA for osteoporosis	Positive	[27]
2014/Taiwan/Lin	Female/77.3 (101/264)	Histology/RUT	DEXA for osteoporosis	Positive in patients with upper GI symptoms	[28]
2015/Japan/Asaoka	Both/63.2 (43/212)	C13 UBT/serum antibody	DEXA for osteoporosis	Positive	[29]

^a^ Number, potential risk for bone fracture patients/control.

DEXA = dual-energy X-ray absorption; RUT = rapid urease test; serum antibody = serum anti–*H*. *pylori* antibody; UBT = urea breath test.

In our study, adiponectin levels were highest in older (age, older than 60 years) postmenopausal women, followed by premenopausal women, and lowest in men. This finding was similar to that of a report by Napoli et al. [26], in which serum adiponectin level was significantly lower in elderly men than in elderly women. Past studies revealed a reverse association between sex hormones (estrogen, testosterone) and adiponectin. Both estrogen and testosterone have a suppressive effect on serum adiponectin [43]. In postmenopausal status, women have decreasing estrogen but increasing adiponectin concentration. Because estrogens can inhibit osteoclast-mediated bone resorption, the positive correlation between osteoporosis and serum adiponectin values could partially be explained by the decreasing estrogen level.

The current study revealed a positive correlation between adiponectin values and 10-year bone fracture probability. This finding was similar to that of some previous studies in which an inverse association between adiponectin and BMD was reported [18, 21]. However, on further regression analysis by adjusting the factors of age and BMI, we could not find associations among adiponectin, leptin, and fracture risk. This revealed a probable confounding effect of body fat and age on the analysis of adiponectin and leptin values. Moreover, recent studies focused on the increase in bone marrow adiposity in osteoporotic patients [44]. Hence, it may be that adipokine level in bone marrow supernatant fluids but not serum total adipokine concentration can discriminate between healthy and osteoporotic status. Further study is needed to determine the importance of adipokine levels in bone marrow fluid to the prediction of osteoporosis outcomes.

Advancing age is an important factor for osteoporosis and bone fracture. In the current study, an age cutoff point of 65.5 years for predicting high bone fracture risk by ROC curve analysis was compatible with the results of previous studies that elderly people (older than 65 years) have highly increased bone fracture risk [4,5,32].

There are some limitations. First, this was a community-based study of more than 2,000 individuals, but the results of BMD were not available for all participants. Hence, FRAX without BMD was applied for the 10-year fracture probability survey. From a Korean study, the FRAX model without BMD appears to show a slightly lower fracture probability compared to that calculated with BMD, especially in younger participants [34]. The current study aimed to evaluate the predictive roles of *H*. *pylori* infection and adipokines for fracture risk. The mean age of participants was about 60 years (not young participants). The application of FRAX without BMD for bone fracture survey is acceptable in this study. The other concern was the short period of surveillance for 10-year bone fracture risk in this study. The outcome of bone fracture did not happen and the fracture risk assessment was uncertain. Second, endoscopy or serum pepsinogen levels were not performed for *H*. *pylori*–related atrophic gastritis evaluation in this study. Because the use of endoscopy is limited in a community survey, no data about gastric mucosal atrophy was available in our study. According to the questionnaire survey, the majority of our participants had no gastrointestinal symptoms. The mean serum calcium values in participants with and without *H*. *pylori* infection showed no significant difference in this study. Whether patients with atrophic gastric mucosa have lower serum calcium levels could not be elucidated in the current study. The last limitation was selection bias. Our study originated from community-based health checkup data. The participants may consist of older adults with underlying diseases and desire for medical examinations. As with the other cross-sectional study designs, unmeasured confounding factors or covariates might exist in this study. In the other way, alcohol consumption survey was performed by questionnaire and no women with high fracture risk reported to drink excess alcohol. People who drank excess alcohol might claim no alcohol consumption in questionnaire. It may be a drawback by questionnaire survey method.

There was no conclusive association among *H*. *pylori* infection, serum leptin values, and bone fracture risk in this study. Although adiponectin was positively correlated with high fracture risk in both sexes, the significant difference disappeared in multivariate analysis by adjusting for the factor of BMI in men group. There may be an existence of co-linearity between adiponectin and BMI in this regression analysis.

## Supporting information

S1 Table STROBE checklist. STROBE checklist for the cross-sectional observational study.
(DOC)
